# 2-Octylcyclopentanone Inhibits Beta Lactam Resistant Diabetic Wound Pathogens

**DOI:** 10.21315/tlsr2023.34.1.15

**Published:** 2023-03-31

**Authors:** Nur Amiera Syuhada Rozman, Tong Woei Yenn, Leong Chean Ring, Syarifah Ab Rashid, Tan Wen-Nee, Jun Wei Lim

**Affiliations:** 1 Universiti Kuala Lumpur, Branch Campus Malaysian Institute of Chemical and Bioengineering Technology, Lot 1988 Kawasan Perindustrian Bandar Vendor, Taboh Naning, 78000 Alor Gajah, Melaka, Malaysia; 2 Universiti Kuala Lumpur – Institute of Medical Science Technology, A1, 1, Jalan TKS 1, Taman Kajang Sentral, 43000 Kajang, Selangor; 3 Chemistry Section, School of Distance Education, Universiti Sains Malaysia, 11800 USM, Pulau Pinang, Malaysia; 4 Department of Fundamental and Applied Sciences, Institute of Sustainable Building, Centre for Biofuel and Biochemical Research, Universiti Teknologi PETRONAS, 32610 Seri Iskandar, Perak, Malaysia

**Keywords:** Beta Lactam Resistance, Diabetic Foot Ulcer, Diabetic Wound Infection, 2-Octylcyclopentanone, Antimicrobial Activity, Ketahanan Beta Laktam, Ulser Kaki Diabetes, Jangkitan Luka Diabetes, 2-Octylcyclopentanone, Aktiviti Antimikrobial

## Abstract

Microbial infection is a frequent complication of diabetic foot ulcers, with up to 82% of ulcers being infected at the initial stage of diabetes. Furthermore, the emergence of beta lactam resistant pathogens managed to eliminate the use of beta lactam antibiotics as a chemotherapeutic alternative. This further increases the amputation and mortality rate. Hence, the aim of this study is to evaluate antimicrobial efficacy of a ketone derivative 2-octylcyclopentanone against diabetic wound pathogens. The inhibitory activity of the compound was determined using disc diffusion and broth microdilution assay. Generally, 2-octylcyclopentanone showed broad-spectrum antimicrobial activity, particularly against beta lactam resistant pathogens. The compound showed comparably better antimicrobial activity than all reference antibiotics, including chloramphenicol, streptomycin, ampicillin and penicillin. In addition, the same compound also inhibits a clinically isolated *Pseudonomas aeruginosa* that was resistant to all reference antibiotics. The activity was microbicidal based on the low minimal lethality concentration recorded, particularly on MRSA, *P. aeruginosa* and *Candida utilis*. The killing efficiency of the compound was concentration dependent. During kill curve analysis, the inhibitory activity of 2-octylcyclopentanone was concentration and time-dependent. 99.9% of reduction of bacterial growth was observed. MRSA and *P. aeruginosa*, two significant diabetic wound infections, are totally inhibited by the molecule at a concentration of minimum lethality concentration. In short, 2-octylcyclopentanone exhibited significant inhibitory towards wide range of diabetic wound pathogens. Which is considered crucial since it will provide a safe and effective alternative treatment for diabetic ulcer infection.

## Abstract (fr)

Jangkitan mikrob adalah komplikasi ulser yang kerap dijangkiti pada kaki pesakit diabetes, yang boleh mencapai sehingga 82% jangkitan pada peringkat awal diabetes. Kemunculan patogen tahan beta laktam berjaya menghapuskan penggunaan antibiotik beta laktam sebagai alternatif kemoterapi. Ini meningkatkan lagi kadar amputasi dan kematian. Oleh itu, tujuan kajian ini adalah untuk menilai keberkesanan antimikrob bagi derivatif keton 2-octylcyclopentanone terhadap patogen luka diabetes. Aktiviti perencatan sebatian ditentukan menggunakan resapan cakera dan ujian cerakinan mikrodilusi. Secara amnya, 2-octylcyclopentanone menunjukkan aktiviti antimikrob spektrum luas, terutamanya terhadap patogen tahan beta laktam. Kompaun tersebut menunjukkan aktiviti antimikrob yang lebih baik berbanding semua antibiotik rujukan, termasuk kloramfenikol, streptomisin, ampisilin dan penisilin. Di samping itu, sebatian yang sama juga menghalang *Pseudonomas aeruginosa* yang diasingkan secara klinikal yang tahan terhadap semua antibiotik rujukan. Aktiviti ini adalah mikrobisida berdasarkan kepekatan kematian minimum yang rendah yang direkodkan, terutamanya pada MRSA, *P. aeruginosa* dan *Candida utilis*. Kecekapan membunuh kompaun adalah bergantung kepada kepekatan. Semasa analisis keluk bunuh, aktiviti perencatan 2-octylcyclopentanone adalah kepekatan dan bergantung kepada masa. 99.9% pengurangan pertumbuhan bakteria diperhatikan. MRSA dan *P. aeruginosa*, dua jangkitan luka diabetes yang ketara, dihalang sepenuhnya oleh molekul pada kepekatan kepekatan maut minimum. Ringkasnya, 2-octylcyclopentanone menunjukkan perencatan yang ketara terhadap pelbagai patogen luka diabetes. Yang dianggap penting kerana ia akan menyediakan rawatan alternatif yang selamat dan berkesan untuk jangkitan ulser diabetik.

**Highlights**
- 2-octylcyclopentanone showed broad spectrum antimicrobial activities, particularly on the beta lactams resistant pathogens.
- The activity was microbicidal based on low minimal lethality concentration recorded, particularly on the MRSA, *Pseudomonas aeruginosa* and *Candida utilis*.
- During kill curve analysis, the inhibitory activity of the 2-octylcyclopentanone was concentration and time-dependent. 99.9% of reduction of bacterial growth was observed.

## INTRODUCTION

Diabetic foot ulcer is one of the major complications of diabetes and it often leads to lower extremity amputation in patients. Lower extremity amputation is common among diabetics (Narres *et al.* 2001). Generally, the wounds of diabetic patients are easily affected once they are exposed to the colonisation of pathogenic microorganism. Wound colonization refers to the multiplying of microbial species on wounds (Kingsley 2001). The microbial communities exist in diabetic wound infection are diverse. They usually exist in a complex polymicrobial biofilm population, which further enhance their survival rate (Peters *et al*. 2012). *Staphylococcus aureus* and *Pseudomonas aeruginosa* are the most common bacteria isolated from chronic wound (Fazli *et al*. 2009; Malic *et al*. 2009; Serra *et al*. 2015).

The management of microbial infection in diabetic foot ulcer patient requires appropriate antibiotic selection. Recently, various types of antimicrobial agents, including heavy metals, natural compounds, synthetic compounds and nanomaterials have been incorporated into wound dressing to promote the recovery of wound (Serra *et al*. 2015). Unfortunately, there is no clear evidence if one option is better than others in treating diabetic food ulcer because the data from previous reports were heterogeneous (Olid *et al.* 2015). The inappropriate and indiscriminate use of antibiotics is a key reason contributing to antibiotic resistance in microorganisms. Silver ions are commonly added in wound care products due to their excellent antimicrobial effects. It has been proven that silver nanoparticles eradicate the biofilm production in *P. aeruginosa* and *S. epidermis* by 98% (Kalishwaralal *et al*. 2010). Despite of this, silver finished product could lead to increased morbidity with chronic ingestions. Moreover, inhalation of heavy metal preparations also leads to deposition of heavy metal particles in the skin, eyes, kidneys and livers (Rovira *et al*. 2015). In other words, due to the exposure or ingestion of excessive silver element, the skin discoloration in wound area appeared which is commonly known as *argyria* (Baker *et al*. 2007). However, it is a challenging task for clinicians to find the most appropriate wound dressing for different types of wounds as no single wound dressing is suitable for all wound treatment.

This study aimed to evaluate antimicrobial efficacy of a ketone derivative 2-octylcyclopentanone against diabetic wound pathogens. Generally, 2-octylcyclopentanone is an alkyl cycloalkanone obtained as a yellow coloured solid with a boiling point of 70°–74°C. The compound has a molecular weight of 196.33, with a molecular formula of C_13_H_24_O. Thus far, the antimicrobial activity of this compound is not reported. However, its derivative, 2-methylcyclopentanone has been reported to exhibit promising antibacterial potential against *B. subtilis* and *E. coli* (Tonari & Sameshima 2000). Due to its unique fragrance, cycloalkanones are widely used as fragrance ingredient in perfumes, soaps and detergents (Scognamiglio *et al.* 2012). The fragrance of *Eucalyptus urograndis* wood is contributed by cyclopentanone (de Souza *et al.* 2018).

In previous study, 2-octylcyclopentanone have been reported as the major compound present in *H. pineodora* essential oil using gas chromatography mass spectrometry (GC-MS) analysis (Rozman *et al*. 2018). The essential oil exhibited excellent antimicrobial activity against test microorganisms isolated from diabetic foot ulcer patients. Therefore, we hypothesised that the antimicrobial activity effect of the essential oil was mainly due to the present of this compound. In this study, we demonstrated the antimicrobial efficiency of 2-octylcyclopentanone against diabetic wound pathogens.

## MATERIALS AND METHODS

### Test Compound

The compound 2-octylcyclopentanone was purchased from Chem-space Company in Latvia. The purity of the compound was 95.0%. The compound was dissolved in 50% methanol to the desired concentration.

### Test Microorganisms

The test bacteria used in this study include 4 Gram-positive bacteria [*Bacillus cereus, Bacillus subtilis, Streptococcus pyogenes* and methicilin-resistant *Staphylococcus aureus* (MRSA)], 6 Gram negative bacteria (*Escherichia coli, Proteus mirabilis, Yersinia* sp., *Klebsiella pneumoniae, Acinetobacter anitratus* and *Pseudomonas aeruginosa*) and 2 yeasts (*Candida albicans* and *Candida utilis*). The microbial strains were previously isolated from diabetic patients in Hospital Seberang Jaya, Penang, with a cohort of chronic wounds (Tong *et al.* 2018). The test microorganisms were sub-cultured on nutrient agar prior to use for every two weeks in order to maintain its viability. The microbial suspensions were prepared by suspending the microbial colonies in sterile physiological saline and the turbidity of each suspension was adjusted according to 0.5 Mc Farland standard.

### Disc Diffusion Assay

Disc diffusion assay was used to screen the antimicrobial efficacy of the essential oil according to a common set of standards created by Tong *et al.* (2014). One hundred microliter of inoculum was streak on the surface of Mueller-Hinton agar (Merck, Germany) by using sterile cotton swap. Then, 6 mm diameter sterile paper discs were then impregnated with 20 μL of 1 mg/mL 2-octylcyclopentanone. The disc was placed on the inoculated agar. Chloramphenicol, penicillin, ampicillin and streptomycin at concentration of 25 μg/mL were included as reference antibiotics to determine the antibiotic spectrum of the test microorganisms. Besides, 20 μL of 50% methanol was also included as solvent control. All plates, including test bacteria and test yeasts, were incubated at 37°C for 24 h. The diameters of the inhibition zone were then measured in mm after the incubation period. The experiments were done in triplicate. The results were expressed in mean of diameter of clear zone ± standard deviation. If no clear zone was observed after the incubation time, the activity was characterised as “resistant.” Otherwise, if a clear zone was detected, the activity was classed as “sensitive.”

### Broth Microdilution Assay

Broth microdilution assay was performed to evaluate the antimicrobial activity of 2-octylcyclopentanone quantitatively (Tong *et al.* 2018). The assay was performed on sterile flat bottom 96-well plate (NEST, Taiwan). All test microorganisms that showed significant inhibitory activity on disc diffusion assay were chosen for this assay. Serial two-fold dilution of 2-octylcyclopentanone was performed with sterile double strength Mueller-Hinton broth (Merck, Germany). After that, 100 μL of microbial inoculum was transferred to each well followed by the addition of 100 μL 2-octylcyclopentanone to achieve a final volume of 200 μL. The mixture resulted in the final concentration of compound ranging from 7.80 to 1000.00 μg/mL. Chloramphenicol was used as a drug control and 50% methanol as negative control in the test. Then, the plate was incubated at 37°C for 24 h. After incubation, 40 μL of 0.2 mg/mL p-iodonitrotetrazolium violet salt (INT) (Sigma, USA) dissolved in 99.5% ethanol was pipetted into each well. The plate was incubated at dark for 45 minutes at 37°C. The colour changed from yellow to purple indicates the presence of microbial growth. The lowest concentration that caused significant microbial growth inhibition was identified as minimal inhibitory concentration (MIC). To determine minimal lethality concentration (MLC) of the compound, the sample in each well was streaked onto Muller Hinton agar plate. After 24 h of incubation, the appearance of microbial colonies was observed. The lowest concentration of test substance that kills the test microorganisms was identified as MLC.

### Kill Curve Study

In order to determine the effect of 2-octylcyclopentanone concentration on bacterial growth, MRSA and *P. aeruginosa* were selected for kill curve study. These two bacteria were selected as test bacteria since they showed lowest minimum inhibitory concentration (MIC) and minimal lethal concentration (MLC) values on broth microdilution assay, and they were identified as important pathogens that cause infection of diabetic wounds. The assay was conducted as per defined by Neta and co-researchers (Neta *et al*. 2016). Initially, 1 mL of bacterial inoculum was inoculated into 23 mL of Mueller Hinton broth. The compound 2-octylcyclopentanone was tested at 4 concentrations: the MIC susceptibility breakpoint, 2 × MIC, MLC and 2 × MLC. A control was included, where 2-octylcyclopentanone was replaced with 50% methanol. The total volume of each flask was 25 mL, and 1 mL of the compound was added to the desired concentration. The cultures were then incubated at 37°C in an incubation shaker. During the incubation period, 500 uL aliquots were taken from each flask at predefined sampling time points (0, 4, 8, 12, 16,..., 40, 44, 48 h). A viable cell count was then performed to estimate the number of live bacterial cells in the sample. The aliquot was plated on Mueller Hinton agar, and the plates were incubated for 24 h at 37°C. The experiment was repeated thrice. The bacterial growth was expressed in logarithm colony forming unit (CFU)/mL of aliquot. The kill curves were plotted versus incubation time and the killing kinetic was analysed mathematically relative to control.

## RESULTS AND DISCUSSION

### Evaluation of Antibacterial Efficiency on Disc Diffusion Assay

The antimicrobial activity of 2-octylcyclopentanone was screened on disc diffusion assay against a wide array of wound pathogens. The results of the assay are presented in Table 1. Generally, 2-octylcyclopentanone showed broad-spectrum of antimicrobial activity against both Gram-positive bacteria, Gram-negative bacteria and yeasts. The diameters of inhibition zones were ranged from 11.0 mm– 21.0 mm. The antibiotic susceptibility spectra of the test microorganisms were tested using both beta lactams (penicillin G and ampicillin), and also aminoglycosides (streptomycin). Based on the results, penicillin G and ampicillin showed only inhibitory activity on one test bacterium, which is *S. pyogenes*. The other test microorganisms were resistant to beta lactams. Besides, 2 Gram-positive and 3 Gram-negative bacteria were sensitive to streptomycin. Chloramphenicol also exhibited broad-spectrum antimicrobial activity on 2 Gram-positive bacteria, 4 Gram-negative bacteria, and 2 test yeasts. Streptomycin and chloramphenicol were used topically to reduce microbial infection on chronic wounds. It is noteworthy that 2-octylcyclopentanone showed comparably better antimicrobial activity than all reference antibiotics. All Gram-positive bacteria and test yeasts were inhibited by 2-octylcyclopentanone. The excellent antimicrobial activity of this compound could be possibly due to its unique mechanism that inhibits the microbial growth. To date, the antimicrobial activity of 2-octylcyclopentanone was not previously reported. Beta lactams were associated with 60% of risk reduction in chronic wounds. However, the efficacy of beta lactams was reduced with the development of multidrug resistant strains of microorganisms. Thus, a higher dosage of antibiotic was needed in order to achieve ideal therapeutic effects.

*P. aeruginosa* is the most frequently isolated Gram-negative bacterium isolated from diabetic ulcers. Besides, this bacterium has the ability to develop resistance to antibiotics rapidly, and this limits the choices of antibiotic treatments. In this study, the clinically isolated *P. aeruginosa* were resistant to all reference antibiotics tested. However, a clear inhibition zone of 13.7 mm was observed with 2-octylcyclopentanone. This reveals the potential use of natural derived 2-octylcyclopentanone in combating multidrug resistant pathogens. *Candida* sp. is the most common cause of fungal infection in diabetic wound ulcers. There were 76.9% of patients underwent amputation due to Candida infection on wound (Öztürk *et al.* 2019). The results showed that 2-octylcyclopentanone inhibited the growth of both *Candida* sp. on disc diffusion assay. The microbial communities exist in diabetic wound infection are diverse. They often exist in a complex polymicrobial biofilm population which consist of different types of microorganisms. Thus, 2-octylcyclopentanone, a natural compound that exhibited broad-spectrum antimicrobial compound is an ideal antibiotic choice to combat diabetic wound infection.

The compound 2-octylcyclopentanone is an alkyl cycloalkanone, which is a type of ketone derivative. To date, no reports are available on the biological activities of 2-octylcyclopentanone. However, the antimicrobial activity of its derivative, specifically 2-methyl-cyclopentanone owned a capability to inhibit *B. subtilis* and *E. coli* (El Sawi *et al.* 2014). Cyclopentanones are frequently isolated from plant essential oil (Huang *et al*. 2009). The aroma of cyclopentanones contributes to the unique fragrance of *Eucalyptus urogandis* (Basak & Candan 2010). Due to this reason, cyclopentanones are widely used as fragrance compound in perfumes, detergents and soaps. Ketone derivatives can be derived from ketones *via* some physical and chemical processes. Many ketone derivatives reported were synthetic organic compounds, and these compounds exhibited significant antimicrobial activity. The antimicrobial activities of plant-derived ketone derivatives were rarely reported. A sesquiterpene ketone, which is known as cyclocolorenone was isolated from *Magnolia grandiflora*, and the broad-spectrum antimicrobial activity of this compound was reported by Jacyno and co-researchers in 1991 (Jacyno *et al.* 1991). Guieranone, a naphthyl ketone derivative isolated from the leaves of *Guiera senegalensis* also inhibited the growth of *Cladosporium cucumenrinum* (Silva & Gomes 2003).

### Broth Microdilution Assay

The MIC of 2-octylcyclopentanone was ranged from 7.80 μg/mL–125.00 μg/mL (Table 2). The wide range of MIC indicates different susceptibility levels of the test microorganisms against 2-octylcyclopentanone. MIC of the compound varied on different test microorganism and the inhibitory activity of the compound was concentration dependent. The MLC of the compound was significantly higher than the MICs. This indicates that a higher concentration of 2-octylcyclopentanone was needed to kill the test microorganisms. In addition, 2-octylcyclopentanone showed the same reading of MIC and MLC against *S. pyogenes*, MRSA, *B. subtilis* and *A. anitratus*. The activity of the compound was bactericidal. It is noteworthy that 2-octylcyclopentanone exhibited significant inhibitory activities to beta lactam clinical isolates. Meanwhile, *C. albicans* was most susceptible to the compound at MIC of 7.80 μg/mL and MLC of 31.25 μg/mL.

In this communication, it is worth to mention a derivative of ketone i.e. 3-methyl-2-cyclopentanone. This compound has been reported to display a low MIC value on *B. subtilis* and *E. coli*, which is similar to our latest outcomes (Tonari & Sameshima 2000). Other ketone derivatives including anthraquinones, naphthoquinones and benzoquinones also showed a significant inhibitory activity with MIC values ranging from 0.002 to 0.128 mg/mL against MRSA (Omosa *et al*. 2016). Beta lactam antibiotics are the most frequently prescribed class of drugs worldwide. These antibiotics inhibit bacterial cell wall synthesis and hence causing the lysis of the bacterial cells (Cho *et al*. 2014). However, the efficacy of beta lactams reduces due to the production of beta lactamase enzyme that hydrolyses the beta lactam functional group. This beta lactamase enzyme causes many failures of antimicrobial chemotherapy since it’s able to convert beta lactam to an inert and ineffective structure (Pence *et al*. 2015). Emergence of these bacteria eliminate the use of beta lactam antibiotics as chemotherapeutic alternative. Hence, the compounds that are able to inhibit the growth of these pathogens are worth researching. *C. albicans* is one of the most common opportunistic fungal pathogens in human. Although it is a normal human microbiome, it also has an ability to colonize human tissues that can eventually lead to several infections (Kuhbacher *et al.* 2017). From the results obtained, 2-octylcyclopentanone possesses a better fungicidal activity on *C. albicans* compared to *C. utilis*. Although they are from the same genus, the drug resistance was different for different species.

### Kill Curve Study

**Figure 1:**
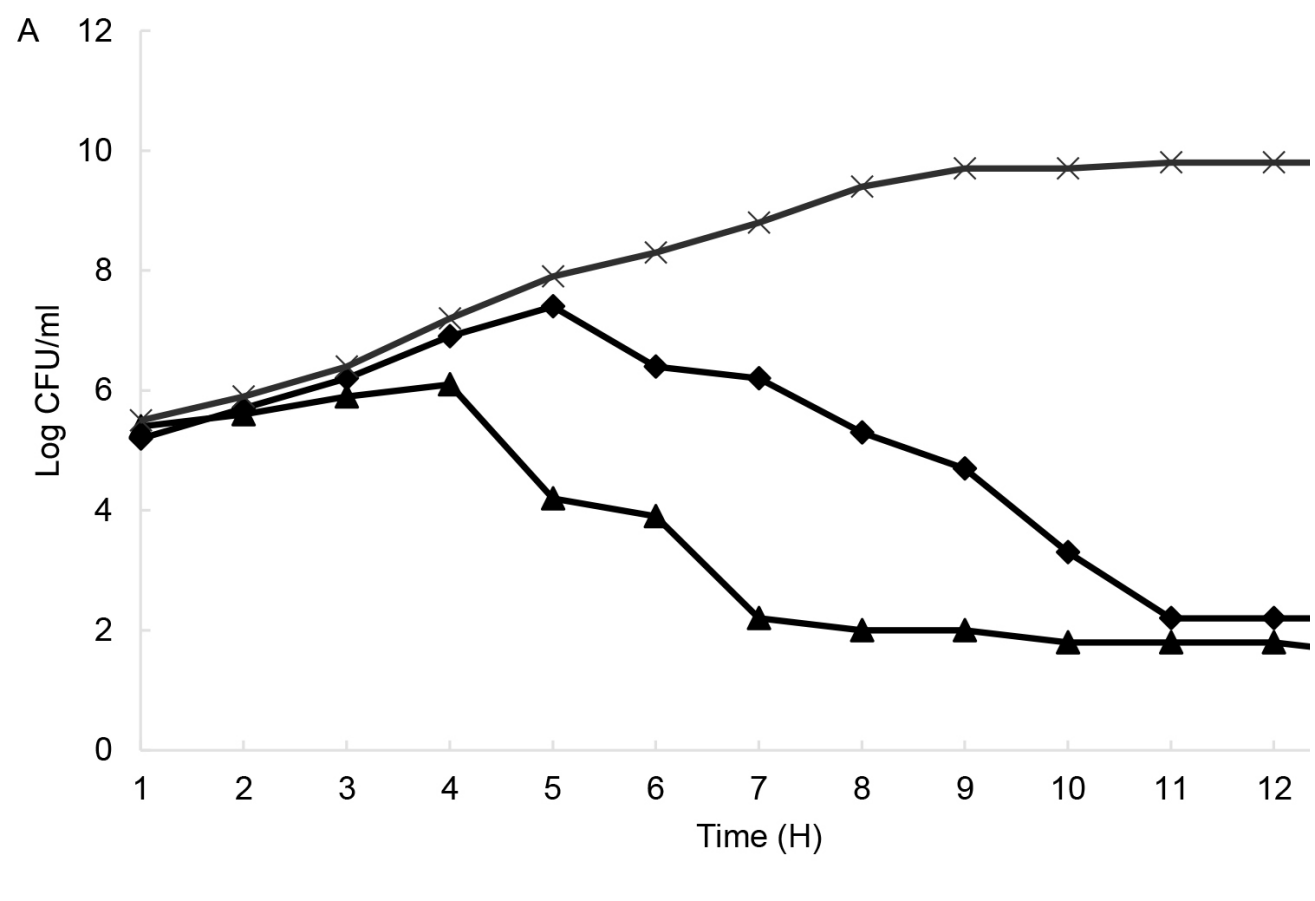

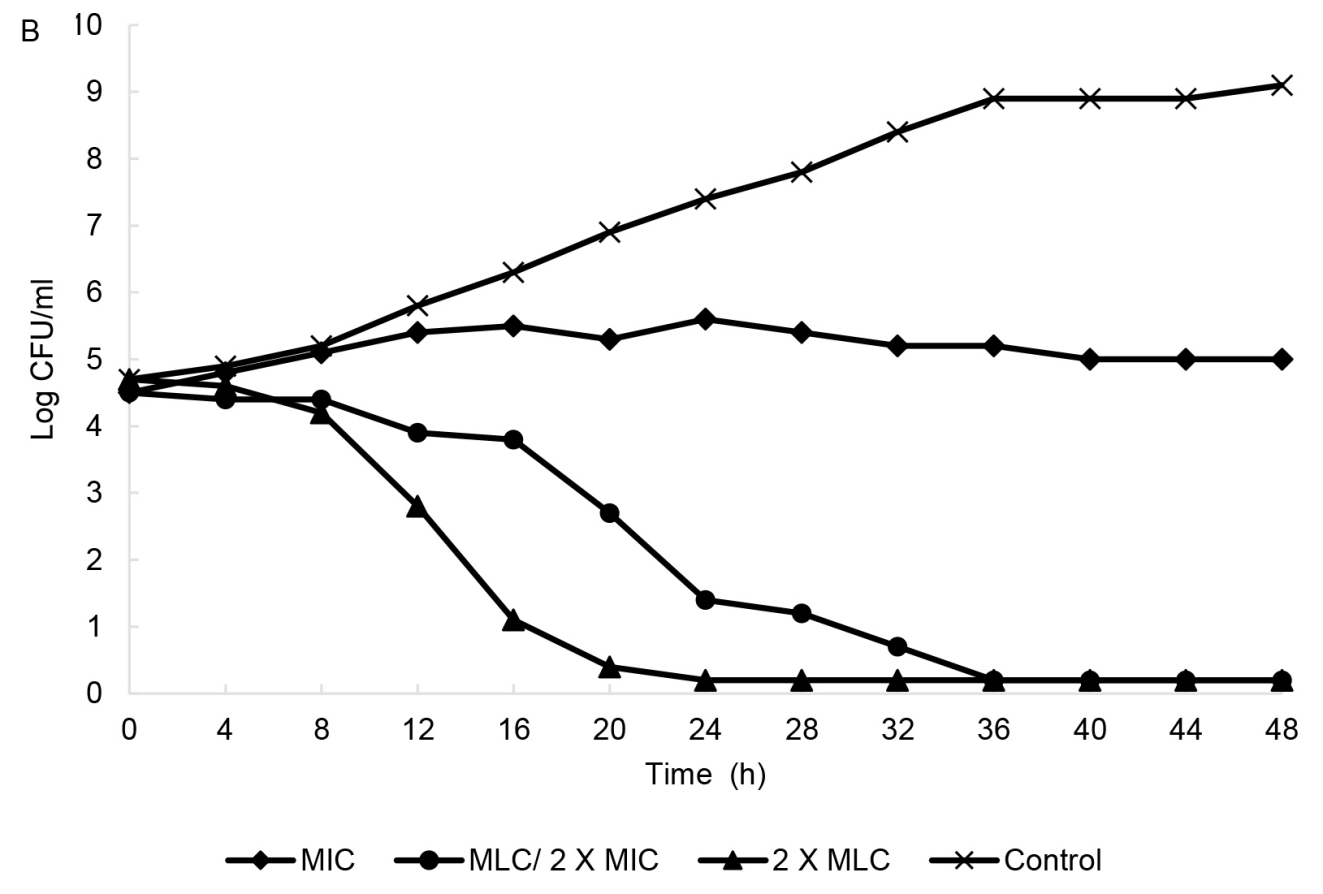
The kill curves of 2-octylcyclopentanone on (A) MRSA and (B) *P. aeruginosa*. The antimicrobial activity was concentration and time dependent.

Figure 1 shows the kill curve of 2-octylcyclopentanone on MRSA and *P. aeruginosa*. These are the two important beta lactam resistant pathogens on diabetic wound. For MRSA, both MIC and MLC were recorded at 25 μg/mL. At MIC/MLC, the bacterial count was significantly lower than control. However, 99.9% growth reduction was only achieved when the concentration of 2-octylcyclopentanone was increased to 2 ×MIC/2 ×MLC. As for *P. aeruginosa*, 2-octylcyclopentanone was bacteriostatic at the concentration of MIC and 2 ×MIC, as 99.9% of bacterial growth reduction was not achieved, relative to control. The kill curves for MIC and 2 MIC were generally similar to that of the control, with a lower bacterial growth observed. At the concentration of MLC and 2 MLC, 2-octylcyclopentanone was bactericidal against both test bacteria.

Viable cell count technique was selected for kill curve analysis as it enumerates only the living cells in the sample (Tong *et al*. 2018). This is crucial to test the bactericidal effect of the test compound. The control growth curves of both bacteria showed 4 distinct growth phases: lag phase, logarithm phase, stationary phase and death phase. The bacterial growth in term of CFU was significantly reduced with the increase concentration of 2-octylcyclopentanone. After 24 h of exposure to 2-octylcyclopentanone, there was a 99.9% reduction of bacterial growth recorded, which relative to control. Moreover, we found out that the higher concentration of test compound was required to kill the microorganisms than to retard the growth of the microorganisms during kill curve study. This finding was in consensus with several reports which using different extracts (Lim *et al*. 2011; Darah *et al*. 2013; Darah & Lim 2015; Tong *et al*. 2017). The kill curve analysis showed that the antibiotic effect of 2-octylcyclopentanone was in a concentration- and time-dependent manner.

## CONCLUSION

In this communication, 2-octylcyclopentanone exhibited broad-spectrum antimicrobial activities against clinical isolates from diabetic wound patients. Therefore, 2-octylcyclopentanone could be a promising treatment for bacterial infection in diabetic foot ulcer patients. Further research should be conducted to elucidate the mode of action of this compound, as the antimicrobial mode of action of alkyl cycloalkanone group of compounds is not reported.

## Figures and Tables

**Table 1:** Antimicrobial activity of 2-octylcyclopentanone and reference antibiotics on wound pathogens.

Test microorganisms	Diameter of inhibition zone (mm)					

		Beta lactam		Aminoglycosides		

	2-octylcyclopentanone	Penicillin G	Ampicillin	Streptomycin	Chloramphenicol	Negative control
Gram-positive bacteria						
*S. pyogenes*	21.0 ± 0.5	7.0 ± 0.1	7.0 ± 0.1	10.0 ± 0.2	10.0 ± 0.2	-
*B. cereus*	11.0 ± 0.3	-	-	-	-	-
*B. subtilis*	12.0 ± 0.4	-	-	-	-	-
MRSA	18.0 ± 0.4	-	-	10.7 ± 0.3	8.0 ± 0.1	-

Gram-negative bacteria						
*P. mirabilis*	-	-	-	15.0 ± 0.6	13.2 ± 0.3	-
*Yersinia* sp.	-	-	-	-	11.4 ± 0.1	-
*A. anitratus*	14.0 ± 0.3	-	-	14.0 ± 0.4	-	-
*E. coli*	10.0 ± 0.1	-	-	-	8.0 ± 0.1	-
*K. pneumoniae*	12.3 ± 0.2	-	-	11.3 ± 0.2	10.1 ± 0.2	-
*P. aeruginosa*	13.7 ± 0.4	-	-	-	-	-

Yeasts						
*C. utilis*	15.0 ± 0.1	-	-	-	10.2 ± 0.1	-
*C. albicans*	15.3 ± 0.2	-	-	-	12.3 ± 0.4	-

**Table 2:** The antimicrobial susceptibility of test microorganisms to 2-octylcyclopentanone on broth microdilution assay.

Test microorganisms	MIC (mg/mL)	MLC (mg/mL)
Gram Positive Bacteria		
*S. pyogenes*	62.50	62.50
*B. cereus*	31.25	62.50
*B. subtilis*	125.00	125.00
MRSA	31.25	31.25

Gram Negative Bacteria		
*A. anitratus*	62.50	62.50
*E. coli*	31.25	125.00
*K. pneumoniae*	31.25	62.50
*P. aeruginosa*	31.25	62.50

Yeasts		
*C. utilis*	31.25	62.50
*C. albicans*	7.80	31.25
